# The need to balance merits and limitations from different disciplines when considering the stepped wedge cluster randomized trial design

**DOI:** 10.1186/s12874-015-0090-2

**Published:** 2015-10-30

**Authors:** Esther de Hoop, Ingeborg van der Tweel, Rieke van der Graaf, Karel G. M. Moons, Johannes J. M. van Delden, Johannes B. Reitsma, Hendrik Koffijberg

**Affiliations:** Department of Biostatistics and Research Support, University Medical Center Utrecht, Julius Center for Health Sciences and Primary Care, PO Box 85500, Utrecht, 3508 GA The Netherlands; Department of Medical Humanities, University Medical Center Utrecht, Julius Center for Health Sciences and Primary Care, PO Box 85500, Utrecht, 3508 GA The Netherlands; Department of Epidemiology, University Medical Center Utrecht, Julius Center for Health Sciences and Primary Care, PO Box 85500, Utrecht, 3508 GA The Netherlands; Department of Health Technology Assessment, University Medical Center Utrecht, Julius Center for Health Sciences and Primary Care, PO Box 85500, Utrecht, 3508 GA The Netherlands

**Keywords:** Epidemiologic research design, Stepped wedge design, Cluster randomized trial, Health economics, Research ethics, Biostatistics

## Abstract

**Background:**

Various papers have addressed pros and cons of the stepped wedge cluster randomized trial design (SWD). However, some issues have not or only limitedly been addressed. Our aim was to provide a comprehensive overview of all merits and limitations of the SWD to assist researchers, reviewers and medical ethics committees when deciding on the appropriateness of the SWD for a particular study.

**Methods:**

We performed an initial search to identify articles with a methodological focus on the SWD, and categorized and discussed all reported advantages and disadvantages of the SWD. Additional aspects were identified during multidisciplinary meetings in which ethicists, biostatisticians, clinical epidemiologists and health economists participated. All aspects of the SWD were compared to the parallel group cluster randomized design. We categorized the merits and limitations of the SWD to distinct phases in the design and conduct of such studies, highlighting that their impact may vary depending on the context of the study or that benefits may be offset by drawbacks across study phases. Furthermore, a real-life illustration is provided.

**Results:**

New aspects are identified within all disciplines. Examples of newly identified aspects of an SWD are: the possibility to measure a treatment effect in each cluster to examine the (in)consistency in effects across clusters, the detrimental effect of lower than expected inclusion rates, deviation from the ordinary informed consent process and the question whether studies using the SWD are likely to have sufficient social value. Discussions are provided on e.g. clinical equipoise, social value, health economical decision making, number of study arms, and interim analyses.

**Conclusions:**

Deciding on the use of the SWD involves aspects and considerations from different disciplines not all of which have been discussed before. Pros and cons of this design should be balanced in comparison to other feasible design options as to choose the optimal design for a particular intervention study.

## Background

**Fig. 1:**
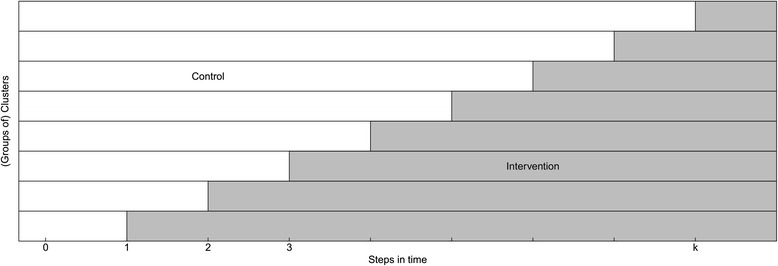
Illustration of the stepped wedge design, where different (groups of) clusters switch from control to intervention at different time points

The cluster randomized trial design (CRT), in which (existing) groups of individuals are being randomized, may be considered when randomization of individual participants is not feasible or desirable [1]. Standard CRTs typically use a parallel design where clusters are randomized to either a control or an experimental intervention for the entire study. Alternatively, CRTs may use a crossover design where at a fixed point in time clusters which started with the control treatment switch to the experimental intervention and clusters which started with the experimental intervention switch to the control treatment [1, 2]. The stepped wedge design (SWD), also called phased or staggered implementation and multiple baseline design [3–5] (though not perceived as exactly the same as SWD [6, 7]), is a special type of the CRT crossover design in which clusters cross over in one direction only [6, 8, 9]. All clusters start with the control treatment after which, at pre-specified time points, one or more clusters switch sequentially to the experimental intervention until eventually all clusters have received the new intervention (see Fig. 1). Within this design, clusters are randomized with respect to the time point at which they cross over (referred to as step), not with respect to a treatment condition or order of treatments as in parallel group or usual crossover designs, respectively. Measurements of the endpoints and other variables of interest are being taken in all clusters during the entire study period. A differentiation can be made between the cohort (longitudinal) and cross-sectional SWD [6, 10, 11]. In a cohort SWD, the same subjects within the clusters are being followed over time, hence the crossover between treatments is then not only at the cluster level but also at the subject level. In the cross-sectional SWD, new subjects are being included after each step, which means that the crossover of treatments is only at the cluster level.

The interest in the application of the SWD in intervention research has sharply increased over the last couple of years [6, 12]. This design was applied in particular to study community level public health interventions that have been proven effective in individual level trials, in so-called phase IV effectiveness trials [6, 9, 12–14], and seems useful for the evaluation of complex healthcare interventions [6, 7, 9, 12, 14–17]. Yet, there is also extensive debate about whether and when the SWD is actually useful for intervention studies [14, 16–22]. The SWD seems to have some natural attractiveness, i.e. the stepwise implementation of the new intervention in each cluster is logistically advantageous [6, 8, 12] and may increase the willingness of clusters to participate [6, 13, 23]. However, this may cause overuse of the SWD, especially when awareness of all potential benefits and drawbacks of this design as compared to feasible alternative designs is fragmented and incomplete.

Previous papers [6, 12, 13] have provided an overview of several perceived pros and cons of the SWD. However, various aspects of the SWD have not yet or only limitedly been addressed, such as considerations related to health economic evaluations of interventions and study ethics. Moreover, discussions of the statistical aspects of the SWD have been limited to sample size and analysis approaches, but do not include interim analyses for example. Furthermore, some pros and cons of the SWD mentioned vary depending on specific characteristics of the study at hand.

Hence, we aim to provide a comprehensive, multidisciplinary overview of the viewpoints on the merits and limitations of the SWD within the CRT setting. We used a set of several methodological papers on the SWD as our starting point to discuss the potential impact of the various features of the SWD in our multidisciplinary team, and to identify features that have not yet been discussed. We set off all identified aspects to the standard parallel group CRT to show which of them are specific to the SWD CRT. Finally, we illustrated several issues by an actual study that applied the SWD. Our overview can assist researchers, reviewers and ethical committees when deciding on the appropriateness of the SWD CRT for a specific intervention study.

## Methods

Within a project team consisting of ethicists, biostatisticians, clinical epidemiologists and health economists (all at assistant, associate, or full professor level), we started off by reading and discussing landmark papers describing the stepped wedge design methodology, and systematic reviews describing in which fields and for which reasons the stepped wedge design has been applied. We performed cross-reference checks to find other papers discussing the merits and drawbacks of this design. Finally, we performed a screen search to assure that we did not miss any key papers discussing the stepped wedge methodology. For this purpose we searched Medline, Embase, Pubmed and Web of Science, using the following phrases: step* wedge*, step* wedge* design, and step* wedge* AND research design. The final search was performed on February 2, 2015 without date restrictions. Articles with didactic purposes or reporting best practice guidelines were also included. Articles only reporting the results of an SWD evaluating a specific intervention, without a methodological focus, were excluded.

Based on the resulting set of articles we identified, categorized and discussed all reported advantages and disadvantages of the SWD in the CRT setting. These served as a starting point for the identification of new, additional benefits, viewpoints, challenges and problems, during a series of informal multidisciplinary meetings. All articles were screened by EH, IT, RG, JR and HK, and decisions on eligibility of articles were made by these authors as well. All found and mentioned aspects of the SWD were discussed and set off to the parallel group CRT to identify which of these aspects are indeed unique for the SWD and whether each aspect is an advantage (+) or disadvantage (−) in comparison to the parallel group CRT. If the consequences of an aspect may be similar for the two designs or their impact may be context dependent this was indicated as (~). The results were categorized into three study phases: 1) the study design and preparation phase, 2) the study execution phase, and 3) the data analysis and interpretation phase.

## Results and discussion

An overview of all identified SWD characteristics and their potential impact is provided in Tables 1, 2, 3 and 4. Table 1 provides key aspects of the SWD. Tables 2, 3 and 4 contain aspects of the SWD in comparison to the parallel group CRT, where Table 2 contains aspects related to the study design and preparation phase, Table 3 those related to the study execution phase, and Table 4 those related to the data analysis and interpretation phase. We will explain certain aspects (indicated with * in the tables) in more detail below.

**Table 1:** Key general characteristics of the stepped wedge design and their implications

Characteristic	Implication
Randomization is usually at the cluster level	Statistical analyses need to take into account that measurements of subjects within a cluster may be correlated
	Concealment of allocation will not always be possible. Blinding of outcome assessment is therefore more difficult to achieve
Cross-over element: each cluster will switch from control to experimental intervention	The cross-over allows for a within-cluster comparison which may increase statistical power
	Sample size calculations as well as analyses become more complex
Two subtypes:	
- switch involves the same patients (cohort-type)	Cohort-type SWD allow for within-patient comparison, which may further increase efficiency, but critical evaluation whether carry-over effects may compromise the results of the study is necessary
- switch involves different patients (cross-sectional type)	
Switch from control to experimental intervention is spread over calendar time	A research team can plan and execute the switch in treatment in a dedicated way as not all clusters switch at the same point in time
	Interim analyses need to take into account that the number of measurements in the control and intervention groups are very imbalanced at early stages and will only be comparable at the end of the study
	It offers the possibility to assess changes in cost-effectiveness over time when the uptake of interventions is difficult or slow due to implementation barriers that need to be overcome
	A study with an SWD may need a relatively long time to complete
All clusters will experience the experimental intervention	This feature may enhance participation of clusters in the study
	The switch in each cluster allows investigation and monitoring of implementation problems
Fixed design in which all clusters start at the same point in time and all steps have the same time span	Preparations for data collection need to be finished in each hospital which can easily delay the start of the study
	Lower than anticipated inclusion rates increase the risk for an underpowered study as solutions like adding more clusters or extending the length of the remaining steps seriously affect the design and are not recommended

**Table 2:** Comparing the SWD to the parallel group CRT: aspects of study design and preparation

Aspect	Issue		Description
Equipoise	~	(2a)*	An SWD may be used in a situation where there is a slight preference for the experimental treatment [6, 12, 13]. At the same time, equipoise remains a necessary requirement for all studies including SWDs. Unlike literature suggests, SWDs where equipoise is disturbed from the start should not be undertaken
† Social value	~	(2b)*	A study with an SWD may benefit fewer individuals after completion since it typically takes longer to complete. However, this disadvantage may be offset by faster implementation following the SWD
† Implemen-tation decisions	-	(2c)*	If evidence on the cost-effectiveness of a new intervention is lacking, collecting this evidence may be valuable to support implementation decisions. However, deimplementation following a negative result has worse consequences for SWDs than for parallel group CRTs
Disease	-	(2d)	An SWD is not the design of choice for a study in a rapidly spreading disease. A pandemic requires an efficient, short-term design and analysis [53]
Study design	~	(2e)	An SWD might be logistically easier because of the phased implementation of the intervention rather than implementation of the intervention at (often) half of the clusters simultaneously in a parallel group CRT [5, 6, 8, 9, 11–13, 16, 34, 54]. However, variations of the parallel group CRT have also been mentioned which give the opportunity of phased implementation [18, 19, 21]
†	+	(2f)	The SWD offers the possibility to assess cost-effectiveness over time when the uptake of the intervention is difficult or slow. Even though statistical power to assess time trends may be relatively low, compared to parallel group CRTs the SWD allows a more accurate assessment of the actual long-term costs and effects after implementation barriers have been overcome
	~	(2g)	An SWD may take longer to complete [5, 6, 9, 12, 13, 16, 18, 53–55]
†	-	(2h)*	In an SWD it will be difficult to compare more than 2 treatments whereas in a parallel group CRT more treatment arms can be added rather easily. Implementing more than 2 treatments may also be of questionable use in an SWD
Sample size	~	(2i)*	An SWD may require fewer clusters than a parallel group CRT [5, 9, 12–14, 16, 17, 21, 34–36]
	~	(2j)*	An SWD may require a larger total number of subjects and/or measurements than a parallel group CRT, depending on cluster size, intracluster correlation (ICC) and number of measurement periods [5, 34, 36]
†	~	(2k)	The effect of incorporating interim analyses on the total sample size for an SWD is not clear yet
Power	~	(2l)	An SWD may have more power than a parallel group CRT due to an increase in the amount of data collected and the possibility of within-cluster comparisons [5, 6, 9, 12, 13, 16–21, 35, 37, 54, 56]
	+	(2m)	The ICC has only a minimal effect on power within an SWD (at least in the cross-sectional design) [9, 11, 13, 35, 48]
Participation	+	(2n)	Clusters may be more willing to participate in an SWD as each cluster will switch to the new (promising) intervention during the study [6, 13, 23]
Timing of outcome	-	(2o)	The time between steps in an SWD should be long enough to detect a treatment effect [5, 9, 12, 13, 18]. Hence, if it takes a relatively long time before a treatment effect can be detected, the SWD may require a much longer time period to be completed than the parallel group CRT

+: positive, −: negative, ~: similar consequences/context dependent, *: discussed in results section, †: newly identified aspect

**Table 3:** Comparing the SWD to the parallel group CRT: aspects of study execution

Aspect	Issue		Description
Informed consent	~	(3a)*	May be difficult to obtain from subjects at the start of the study [13], and both SWDs and parallel group CRTs need modified informed consent procedures [57]
			In cross-sectional SWDs the informed consent is in essence similar to that of a parallel group CRT. In cohort SWDs participants will have to understand that the moment of receiving the new intervention is being randomized
Study participation	-	(3b)*	An SWD may have increased risk of drop-outs and drop-ins (contamination) [6, 13, 18, 21, 22, 54]
† Inclusion rate	-	(3c)*	An SWD suffers relatively more from low inclusion rates because adding a cluster or extending the steps during the trial disrupts the symmetry of the design
† Study duration	~	(3d)	The possible longer study duration of SWDs might require interim analyses to avoid long exposure of clusters of participants to suboptimal care when the new intervention would be clearly inferior/superior to usual care. The statistical analysis aspects of interim analyses in an SWD are, however, still unclear
† Number of measurements	-	(3e)	If collecting data on health outcomes or costs is expensive, it may not be feasible to collect health economic evidence at each time point (step) in a cohort (longitudinal) SWD. This is particularly relevant if the number of steps (and hence number of measurements per participant), would be large. Even though a similar parallel group CRT would require more participants it might require fewer measurements in total [5, 34–36], and therefore could be more feasible
	-	(3f)	Repeated measurements within the SWD may lead to a higher burden on everyone involved in the study. In the cross-sectional setting, this will not be a problem for individual participants, but may still be for research personnel [13, 16, 18, 19, 22, 35]
Blinding	-	(3g)	Blinding of participants and care providers is often impossible within SWD, however this also holds for the parallel group CRT. Hence, blinding of assessors of the outcomes is advised [6, 11–13, 19]
Improving intervention	~	(3h)	Within the SWD it is possible to improve the intervention during the study, though it is questionable whether it is desirable to do so [12, 18, 22, 56]

+: positive, −: negative, ~: similar consequences/context dependent, *: discussed in results section, †: newly identified aspect

**Table 4:** Comparing the SWD to the parallel group CRT: aspects of data analysis and interpretation

Aspect	Issue		Description
Effect estimate	-	(4a)	In an SWD, the unidirectional crossover strategy complicates the statistical analysis [6, 9, 11–13, 36]. Any temporal trends or fluctuations may (partially) invalidate the statistical analyses used by default [36]. If temporal trends or fluctuations are expected or found, a simple within-cluster analysis can provide a biased estimate of the treatment effect [9]. Calendar time is a potential confounder and should be adjusted for in the analysis [37]. Since incorporation of the effect of time requires a modelling approach, assumption-free analysis methods (nonparametric methods) cannot be used to analyse SWD data
	+	(4b)*	In an SWD the effect measure of interest (e.g. difference in means or relative risk) can be calculated for each cluster, and the (in)consistency in effect estimates across clusters can be examined [37]
†	+	(4c)	In an SWD learning and decay effects over can be assessed over time, i.e. due to more experience with the intervention outcomes may become better over time. However it could also be that the intervention is well adopted just after implementation but ‘forgotten’ about after some time (e.g. if the intervention consists of new guidelines)
† Interim analyses	-	(4d)*	Interim analyses within an SWD are less efficient due to the unequal numbers of measurements under the different treatment arms during the study. For parallel group CRTs these numbers are more comparable during the entire trial period
† Number of measurements	+	(4e)	Collecting evidence on outcomes at several time steps may allow assessment of the (changes in) these outcomes during a longer follow-up period in those clusters that crossed over early in the study. This might benefit subsequent statistical and health economic analyses, for example, when extrapolating beyond the trial horizon
† Unrelated studies	+	(4f)	Collecting health economic evidence in an SWD might also provide insight into general barriers and facilitators to implementation and into changes in cost-effectiveness when moving from a clinical to a routine care setting. In an SWD more evidence on implementation is collected than in parallel group CRTs, as the process of implementing the new intervention can be observed during the study, for all clusters, as opposed to parallel group CRTs where half of the clusters do not get the intervention during the study, and studying changes in implementation over time is more limited. This additional evidence might be valuable in the design and execution of other studies, for example, studies on other interventions in the same disease area

+: positive, −: negative, ~: similar consequences/context dependent, *: discussed in results section, †: newly identified aspect

### Study design and preparation phase (Table 2)

In comparison to the parallel group CRT design, the SWD raises two ethical challenges. Although clinical equipoise and social value are ethical requirements for both designs, the content of these requirements may substantially differ in the two designs, as we will explain below.

#### Clinical equipoise (2a)

It has been argued that there should be “genuine uncertainty in the expert medical community about the preferred treatment” before a randomized trial is allowed to be conducted [24]. Ensuring this uncertainty balances the duties of physicians as caregivers and researchers. If there is clinical equipoise among two (or more) treatments, physicians who are researchers do not violate their therapeutic duties by withholding a (possibly superior) treatment from their patients.

The SWD is frequently used in situations where the intervention under study has “shown to be effective in more controlled research settings” or may not have shown to be effective yet but is strongly believed to do more good than harm [6, 12]. At first sight, clinical equipoise seems to be absent in these situations. At the same time, one may argue that in spite of the lack of equipoise all participating clusters will receive the seemingly superior experimental intervention at some point during the study. This assumption can be questioned: one should note that although all *clusters* will receive the experimental intervention, it does not always mean that all participating *subjects* will receive the experimental intervention. In a cohort design this is the case, but in a cross-sectional SWD only half of the participants will receive the experimental intervention just as in a parallel group CRT [16]. Thus the SWD cannot always prevent that some participants are withheld from the seemingly superior intervention.

Yet it still remains to be proven whether the experimental intervention that is felt superior is actually better. An intervention might have shown efficacy (i.e. work under controlled settings), though still really needs to be evaluated for effectiveness (i.e. does it work in practice). Moreover, when both the experimental intervention and the control arm are established effective interventions participants are in principle not withheld from care as usual, even when one of these arms is felt to be slightly superior before the start of the study. At the same time, when the risks of the arms substantially differ this must be disclosed to research participants [25].

#### Social value and health care decision making (2b and 2c)

Social value implies that research should be conducted with the aim to produce generalizable health knowledge that will ultimately improve the health of individuals and/or the public [26]. This is an important ethical requirement for human subjects research since the resources for research are limited, so should be well-spent, and people should not be put at risk for the benefit of science and society if there is no social value to be expected [26]. Although results obtained from a singular study using an SWD may lead to health benefits, the social value of SWD studies may be limited compared to parallel group CRTs, in particular from a health economic perspective. Given a limited research budget, performing one study effectively prohibits the execution of another study. This is known as the opportunity cost of a study and represents the “cost” incurred by not enjoying the benefits (i.e. social value) from the best, alternative research activity with similar resource costs [27]. The benefits of performing a study can be defined as the additional insight gained into the health effects, costs and cost-effectiveness of the experimental intervention compared with the control treatment. However, this insight is only valuable when it actually improves decision making on whether to adopt the experimental intervention immediately, to adopt it while also requiring additional evidence collection, to adopt it only in research settings in order to collect more evidence, or to reject the experimental intervention [28]. Such policy decisions are partially based on the balance between the monetary costs and the resulting health benefits of the experimental intervention, i.e. cost-effectiveness. Therefore, the social value of studies is largest when the cost-effectiveness of an experimental intervention is highly uncertain, such that additional evidence may largely reduce the risk of making wrong policy decisions.

The following two situations illustrate the limited usefulness of the SWD from a health economic perspective. First, if the cost-effectiveness of the experimental intervention is highly uncertain, new and unfavourable evidence on health outcomes and costs may lead to the rejection of the experimental intervention. Such a rejection decision might be costly since all clusters will have implemented the intervention by the end of the SWD study. Indeed, all clusters have invested but now need to disinvest while the investment costs may be irrecoverable. On the other hand, if the decision to implement will not be affected by new findings, collecting additional evidence on health outcomes and costs within an SWD is inefficient because this evidence will not influence policy decisions anymore and therefore will have no social value. Although this holds irrespective of whether a parallel group CRT or SWD design is being used, the higher disinvestment costs makes this situation more likely for SWD studies.

Health economic methods have been developed to estimate the social value of a study [29, 30]. These, so-called, value-of-information methods quantify the expected improvement in health outcomes and expected changes in health care costs when the adoption decision can also include evidence from the new study instead of including existing evidence only [31]. The expected social value of a new study increases when the additional evidence it collects will benefit more individuals in the future (that is, when many individuals are eligible for the experimental intervention) and when the study rapidly delivers evidence that remains valuable for a long period of time. Conversely, when some individuals would already receive the experimental intervention the social value of a new study decreases because treatment would change for fewer individuals. In particular, only those future individuals that would have received the control treatment might benefit from a switch to the experimental intervention. In general, these issues might lead to a lower social value of an SWD study as compared to a parallel group CRT, as the SWD study may take longer to complete which reduces the number of future individuals eligible for the experimental intervention. This reduction could, however, be compensated by a faster total implementation process in the entire region of interest (e.g. country or state) following study completion for SWD studies as compared to parallel group CRTs. This advantage depends on the number of clusters included in the SWD or parallel group CRT and the number of not included clusters in the region of interest. If the experimental intervention is accepted for use in clinical practice, the number of clusters in which it still needs to be implemented can be substantially lower after SWD studies than after parallel group CRTs.

#### Study design (2h)

In a parallel group CRT more than two treatment arms can be included rather easily. For example, usual care can be compared with two experimental interventions within the same study, as illustrated by de Smet et al. [32]. Within the SWD however, this is more difficult. One question that arises is how the clusters will cross over from control to the experimental interventions since there are two possibilities to do so. First, the experimental interventions can be implemented sequentially, that is, clusters cross over from control to one experimental intervention first and at a later time point (step) cross over from this intervention to the other experimental intervention. Secondly, the cross over to one of the experimental interventions can take place at the same time, that is at one step some clusters cross over from control to the first experimental intervention while other clusters cross over to the other experimental intervention. In case of the first situation a (much) longer study period may be required than in the latter situation. In addition to this practical question, it is unclear what the appropriate methods are to calculate the required sample size and to analyse the data resulting from such a three-arm design. Moreover, the question is whether the SWD is a suitable design given the aim of a three-armed study. If there is no practical need to implement the treatments sequentially in clusters over time, other designs will probably provide results in a shorter period of time than the SWD and will therefore be preferable. If, on the other hand, the experimental treatments consist of training of professionals for example, the sequential implementation within the SWD may be very attractive upfront. However, in this example the question is whether the effects of the experimental interventions do not carry over within a cluster from one period to another (given that the trainings are substantially different from each other). One exception to this proposed situation is when one intervention is an add-on to another intervention. For example, in the Helping Hands study [33] one intervention targeted the individual professionals and included education, reminders and feedback. The other intervention extended this by targeting the team level as well and focussed on social influence in groups and leadership. In this example, sequential implementation could be a reasonable choice and carry-over effects would not be an issue. However, since all clusters will be trained for one or both of the experimental interventions, the SWD will probably be more expensive than other design options. Given these issues, it seems questionable whether an SWD is useful if more than two treatment arms are to be included in one study.

#### Sample size (2i and 2j)

The SWD is often thought to be more efficient than the parallel group CRT since it uses both within- and between-cluster comparisons to estimate a treatment effect [9]. However, in the comparison of designs a difference should be made whether efficiency is in terms of the number of clusters, the number of participants or the total number of measurements required. Furthermore, a difference should be made between a cohort and a cross-sectional design. At the moment, only sample size and power formulae are available for the cross-sectional setting, hence the following discussion will be limited to this setting. Finally, a clear definition of what the parallel group CRT entails should be taken into account. If the parallel group CRT includes only one follow-up measurement of each participant in the analysis (i.e. one measurement period of *m* participants per cluster), then given an equal cluster size *m* per measurement period for the SWD, the SWD always requires fewer clusters than the parallel design [34, 35]. However, whether in this case the SWD is also more efficient in terms of the number of participants (which equals the number of measurements in a cross-sectional setting) depends on the cluster size, intracluster correlation coefficient (ICC), and the number of steps [34, 36]. In general, if the ICC is small, the parallel group CRT will have more power, whereas the SWD will have more power in case of a large ICC [37].

If the analysis of a parallel group CRT includes a baseline measurement as a covariate (resulting in an analysis of covariance), then the SWD does not necessarily require fewer clusters in comparison to a parallel group CRT with three measurement periods (one baseline and two follow-up) as shown by Rhoda et al. [5]. They showed that, given a cluster size of 100 participants per group per measurement period, if the ICC is rather small (<0.005) and the number of steps is up to three, the SWD requires more clusters. In case of four steps and ICC ≥ 0.005 or at least five steps and ICC ≥ 0.0001, the SWD requires fewer clusters. However, the total number of measurements (and hence participants in a cross-sectional setting) is higher for the SWD in most cases, except for situations where the ICC ≥ 0.05 and the number of steps is between five and eight. Note that when the cluster size changes the cut-offs in ICCs and numbers of steps will change as well since all of these factors affect the required number of clusters.

Although the numbers might change slightly when the third measurement would not be taken into account as in [5], it can still be expected that the SWD will not always require fewer clusters nor participants depending on cluster size, ICC and number of steps. Yet, Hemming et al. [37] showed that irrespective of the value of the ICC, an SWD with four or more steps will have more power than a parallel group CRT that includes a baseline period (i.e. a two measurement periods design).

Since the SWD requires fewer clusters than the parallel group CRT in many cases as described above, the SWD is especially advantageous when the number of available clusters is limited [12, 14, 16, 21, 22, 34]. Yet, one should be aware that this may come at the cost of a higher number of required participants/measurements.

### Study execution phase (Table 3)

#### Informed consent (3a)

Thus far only Zhan et al. [13] have identified informed consent as a key issue in SWDs by noting that the timing of the informed consent procedure can be difficult. In their trial, the Research Ethics Committee considered it unethical to ask patients informed consent at the start of the study (when care as usual was delivered). Unfortunately, it remained unclear what the exact reasoning was for this judgement but some possible reasons can be listed.

In individual randomized trials, informed consent is given at once for data collection, randomization and administering the experimental intervention, whereas in CRTs participants have to give separate informed consent for these three elements [38]. In particular the latter two elements may raise ethical issues. In CRTs, often the randomization of the clusters has taken place before participants enter the cluster, for example, a hospital has been randomized to one of the treatment arms before a patient enters. Besides, it is usually impossible for participants to move to another cluster and therefore it is then almost virtually impossible for a patient to opt-out (for instance in an emergency situation as in the example of the HEART study described below). This means that participants in CRTs need to consent that they have already been randomized rather than going to be randomized to one of the treatments. These issues are true irrespective of the design choice (parallel group or SWD).

Yet, the informed consent for randomization in an SWD differs from the parallel group CRT in the sense that in a parallel group CRT a cluster is either randomized to the control or the experimental intervention whereas in an SWD all clusters will start providing the control intervention but will switch to the experimental intervention at some point during the study. In a cross-sectional SWD new participants are included after each step leading to half of the participants being exposed to the intervention and the other half to the control. Then the informed consent process is not different from a parallel group CRT. However, if the same participants are being followed over time (a cohort SWD) participants must understand that they are not randomized to one of the treatment arms but that the time point at which they will switch from the control to the experimental treatment is being randomized. So, although participants in this situation will receive the experimental treatment at some time during the study, they should understand that it could be shortly after the start of the study but that it could also be almost at the end of the study. Disclosure of this information may increase the risk of contamination.

#### Study participation (3b)

Although it is likely that clusters are more willing to participate in an SWD than in a parallel group CRT (because they know they will receive the experimental intervention somewhere during the study) [6, 13, 23], drop out of clusters could be more likely as well. Especially when clusters are being randomized to later steps (which they should not know in advance), they might drop out just because of this delay (clusters may lose interest [21]) or because a similar kind of intervention as the experimental one becomes available during the study. Drop-out of participants will not be an issue if new participants are being included after each step (cross-sectional design). However when the same participants are being followed over time (cohort design) there will be an increased risk of drop-out similar to other longitudinal research designs.

On the other hand, drop-ins may also be more likely in SWDs than in parallel group CRTs [18]. Drop-ins may occur at the participant level in two ways: a participant may already receive the experimental treatment from his caregiver while this caregiver should give the control treatment by design, or the participant may move to another cluster that has already switched to the experimental treatment. The first type of drop-in could be considered a non-adherent cluster, which could lead to biased (contaminated) results if such a participant would be included in the analysis as if he were in the control intervention. The effects of the second type of drop-in may have only limited effect on the results since such a patient then becomes a member of a different cluster and his outcomes would be considered to belong to the ‘new’ cluster. Only if this patient would still report outcomes to his initial cluster as if he received the control intervention, bias may occur. These types of drop-ins can occur in parallel group CRTs as well, but are more likely in SWD studies because they often take more time to complete. Drop-ins at the cluster level are less likely, since clusters agreed with the stepwise implementation within the study upfront.

#### Inclusion rate (3c)

The effect of lower than expected inclusion rates can be very detrimental in SWDs, because it will disturb the balance in the design and will cause a loss of power. Whereas in parallel group CRTs the inclusion period can be extended rather easily, this is not straightforward in SWDs since it would mean that the time between steps needs to be prolonged which may result in a much longer study period than planned beforehand. This may not be feasible due to financial or other constraints. Furthermore, such a change during the study will result in different lengths of time and different numbers of measurements between steps. If, and how, this may affect the results and validity of the study is not clear yet. Moreover, if the inclusion rate lags behind and it is therefore decided to prolong the time between steps, the risk of drop-out may increase due to further delays for clusters not yet receiving the experimental intervention.

### Data analysis and interpretation phase (Table 4)

#### Measure of effect in each cluster (4b)

Because each cluster in an SWD will switch from control to intervention, it is possible to calculate the effect measure of interest (e.g. difference in means, relative risk, odds ratio) in each cluster and examine the consistency in effect across clusters [37]. Tools applied in meta-analysis can be used to visualize (forest plot) and quantify the inconsistency (τ^2^ and *I*^*2*^ as heterogeneity measures). Consistency in effect across clusters may increase the strength of the overall finding, whereas inconsistency may complicate the interpretation of the overall finding [39]. Reasons for inconsistency may be explored. Even though the design is often not powered for these analyses, they may still provide additional insights [37].

#### Interim analyses (4d)

Cumulative monitoring of a study might be warranted to detect potential harmful effects or early dramatic benefits of a new intervention. Several group sequential methods are available and extensively studied for individual randomized trials, but little is known about the application of these methods in the CRT setting. It has been shown that the Pocock and O’Brien-Fleming boundaries can be applied to parallel group CRTs with a binary outcome in order to control the type I error rate, given that all clusters are recruited simultaneously (i.e. start the trial at the same time) and individuals are recruited from those cluster sequentially [40]. However, the aspect of adjustment of the effect estimates following a group sequential CRT has not been studied.

Given that the SWD is most often used when the intervention is thought to do more good than harm and in implementation research, stopping early for harmful effects is unlikely. However, beneficial effects may lead to early stopping and speeding up implementation in the clusters which were allocated to switch treatments at later.

The cross-sectional SWD will use the same recruitment pattern as described for the parallel group CRT. Hence, the results presented above can be expected to apply to SWDs as well. However, within the SWD unequal numbers of measurements are available for each condition after each step (with exception of the last period). That is, at the start of an SWD only measurements under the control condition are available. Then from the first step onwards, the inequality between the number of control and experimental intervention measurements becomes smaller, until at the end of the study the number of measurements under each intervention is similar. The effects of these inequalities on the statistical analysis and power at interim time points are yet unclear, though the unequal numbers of measurements under each treatment over time will generally result in a loss of efficiency [5, 41].

## Example: the HEART impact study

Several aspects of the SWD will be illustrated by assessing the HEART Impact study [42–44]. This study uses a cross-sectional SWD and the inclusion of this study has recently completed. Patients presenting at an emergency department with chest pain pose a challenge to physicians since chest pain can be the symptom of an acute coronary syndrome (i.e. acute myocardial infarction or unstable angina) requiring prompt treatment. However, in up to 80 % of the patients it is caused by another, usually non-life-threatening condition. The HEART score has been specifically developed to stratify patients with chest pain according to their risk for cardiac events. Previous studies have shown that this score can adequately identify patients with low, medium or high risk for developing cardiac events [43, 44]. A few hospitals in the Netherlands already apply the HEART score, though other clinical prediction rules [45–47] are being used as well. Yet, the majority of hospitals (or treating physicians) do not use any of the formal prediction rules.

The aim of the HEART Impact study is to quantify the impact of the active use of the HEART score in daily practice on clinical decision making, patient outcomes (incidence of major adverse cardiac events within 6 weeks and quality of life), use of health care resources and costs. The hypotheses are that the use of the HEART score will be safe (comparable incidence of major adverse cardiac events when using the HEART score compared to usual care), will improve the management of the patients presenting with chest pain (in particular fewer hospital admissions and additional testing in low risk patients when using the HEART score), and will reduce overall healthcare costs in this group of patients.

Table 5 shows which aspects of the SWD are relevant in this study. Some of these aspects will be explained in further detail below.

**Table 5:** Key aspects of the stepped wedge design in the HEART study

Aspect	Issue	Description
Implemen-tation decisions	(5a)*	Based on the results of previous validation studies it is likely that the HEART score will be cost-effective if applied correctly (including adherence to management recommendations). The SWD has the benefit to demonstrate the value of the HEART score in real practice and problems in implementation can be observed and documented in each cluster
	(5b)*	When a formal decision would be made on nation-wide implementation of the HEART score based on cost-effectiveness estimates from the HEART Impact study, the costs of disinvestment (de-implementation) have to be considered. As the intervention under investigation is the use of a clinical prediction model disinvestment costs are likely to be very small and not much larger in the SWD than they would have been in a parallel group CRT design
Equipoise	(5c)	Earlier validation studies have demonstrated the ability of the HEART score to stratify patients with chest pain according their risk of having a serious heart condition. However, it is unclear whether actively using the HEART score in practice will indeed be safe and improve health care in terms of health care resources, patient burden and costs
Participation	(5d)	The use of risk scores in chest pain patients is recommended in (Dutch) guidelines. The SWD was attractive for hospitals as each hospital would experience using the HEART score during the trial
Preparation	(5e)	Inclusion in the HEART study started almost a year later than planned as all hospitals need to start at same time. Procedures in 1 hospital were slow, which contributed to the delayed start
Informed consent	(5f)*	No informed consent from patients was sought to determine HEART score
		Informed consent was asked from patients to collect additional data
		Timing of consent: during the initial evaluation by the treating physicians at the emergency department
Study design	(5g)	A mix of hospitals (with respect to size, city and rural, academic and non-academic) participates in the HEART study leading to differences in population and standard of care between hospitals. The SWD allows for a within-hospital comparison reducing the impact of these differences
	(5h)	A mix of hospitals participates in the HEART study leading to noteworthy variation in numbers of included patients per hospital which has not been taken into account in the sample size calculation
Blinding	(5i)	The primary outcome is major adverse cardiac events (MACE), which has some subjective elements. There will be an adjudication committee blinded for intervention period for the main endpoints
Interim analyses	(5j)	The HEART study has been classified as a low-risk trial. Therefore, no formal interim analyses are planned. A DSMB is monitoring the trial in particular to give an independent advice to participating hospitals about continuing the use of the HEART score at the end of the study only
Sample size	(5k)	Inclusion rates have been much lower than expected. The study team considered adding clusters or time points to the study, but decided not to do this because it is unclear how to accommodate such changes properly in the final statistical analysis. Furthermore, there was considerable uncertainty about the assumptions in the initial sample size calculation
Method of analysis	(5l)	A Generalized Linear Mixed model (GLMM)-analysis is planned to take into account the hierarchical nature of the data
	(5m)	No interim economic evaluation has been planned. Negative results in the health economic analysis could, at least in theory, lead to de-implementation of the HEART score. As this process requires time and money, depending on the number of hospitals already switched to HEART, performing a preliminary economic evaluation as part of an interim analysis might have been worthwhile
	(5n)	The (in)consistency in effect across clusters (hospitals) will be examined in a explorative way, for instance whether the effect size varies depending on type or size of hospital

*: discussed below

### Implementation decisions (5a and 5b)

Collecting data on health outcomes and costs in the HEART Impact study will allow assessment of the cost-effectiveness of the HEART score compared to usual care in clinical practice. Cost-effectiveness results are likely to provide crucial input to a subsequent formal decision on whether to implement the HEART score in the entire Netherlands. Indeed, the HEART-Impact study is expected to provide definitive evidence on the (favourable) cost-effectiveness of the HEART score, meaning that additional studies would have no social value. In the (unlikely) event that the collected evidence would contradict the expected safety and cost savings, de-implementation of this specific intervention in all participating hospitals is very straightforward and virtually costless. Consequently, the collected data on cost-effectiveness is valuable and likely to be used in decision making on (further) implementation of the HEART score.

### Informed consent procedure (5f)

In general, informed consent procedures in CRTs, whether parallel cluster or stepped wedge, are ethically complex (see above). Obtaining informed consent also raised ethical issues in the HEART Impact study, in particular because the intervention under evaluation consists of two steps. The first step is to determine the HEART score based on routinely collected information directly after presentation. The researchers of the HEART study received a waiver of informed consent from the research ethics committee for calculating the HEART score since this score is evidence-based, part of the medical professional standard, and determining the score does not involve additional, invasive procedures. The second step of the HEART intervention is the link between the height of the HEART score and certain management options, for example the suggested management for patients with a low HEART score is discharge to home. In practice, there was a time interval between the first and second step of the intervention. During this time interval, the purpose of the study was explained and informed consent for additional data collection and follow-up was obtained. In theory, there were two ways in which patients could opt out: (a) patients refuse consent for data collection and for using the HEART score to guide further treatment decisions; (b) patients refuse consent for data collection but not for using the HEART score to determine further treatments. At first sight, the latter situation seems unproblematic since physicians do not have to consider alternative strategies to guide further treatment decisions. However, the information that is present is essentially the same as in the first situation. Patients who are given the opportunity to opt out are in a situation where it is almost impossible to *meaningfully* opt out, as we set out earlier in the ethics section since they cannot go to another hospital. Moreover, if they choose to opt out and hence to receive care as usual, this care as usual will be based on the clinician’s professional judgment, which could be guided by the HEART score, by an alternative risk score that the physician used to apply previously, or by his or her overall risk assessment without formal prediction rules.

## Conclusions

Our aim was to provide a comprehensive overview of the pros and cons of the SWD from a multidisciplinary viewpoint, moving beyond a systematic review only reiterating previously discussed aspects. We showed that many aspects from different disciplines need to be considered when deciding on a SWD, not all of which have been discussed before. If researchers consider using an SWD for their intervention study all these aspects should be taken into account since seemingly attractive aspects may be outweighed by negative or yet unclear effects of other aspects.

Whether the SWD is the best design option for a specific intervention study needs to be decided by considering all, multidisciplinary (statistical, methodological, ethical and health economical) aspects in comparison to other feasible designs. We agree with Hemming et al. [37] that the SWD is likely to be preferable for studies where some evidence of effectiveness of the experimental intervention is already available, or in cases where the intervention is a service delivery or policy change that does not need individual informed consent to be implemented and where the outcome is preferably available from routinely collected data. However, the SWD may not be a good choice in case of a high risk intervention for which effectiveness has not been shown yet, or in case of an intervention unlikely or unfeasible to be de-implemented when proven ineffective.

Several variations on both the simple SWD and parallel group CRT are possible, such as the staggered parallel group CRT and incomplete SWDs [18, 48]. Although we did not take these variations into consideration explicitly, many of the addressed aspects will still be an issue, though possibly to a different extent.

We distinguished between cross-sectional and cohort designs throughout this paper. For many aspects one can easily see the potential differences. However, for power and sample size calculations this is not straightforward. The available approaches all rely on the cross-sectional design [5, 9, 35, 48, 49]. If a cohort or a mixture of the cross-sectional and cohort design is to be used, both sample size calculations and analyses need to take into account the correlation over time not only at the cluster but also at the individual level. Furthermore, changes over time at both levels should be taken into account. However, it is not clear yet how to incorporate these factors into the design and analysis of SWD studies. Although cohort designs are more efficient than cross-sectional designs in general [50–52], future research is required to see whether this also holds for the SWD (given variations in ICC, number of clusters, cluster size and number of steps) before further comparisons can be made with other designs in this respect.

Several other aspects have been noted to require further research. For example, methods for sample size and power calculations so far have been limited to cross-sectional designs and assumed equal cluster sizes between both clusters and steps. Besides, the possibility of including more than two treatment arms is questionable. Finally, little is known about the use of interim analyses in CRTs altogether [40]. Given that one might expect to be able to stop early for effectiveness within an SWD in situations where there is already an indication of superiority of the new intervention, it is important to know how to perform the interim analyses properly.

In summary, we have provided an overview of all aspects of the SWD that should be taken into consideration when a choice will be made between this design and other valid design options. The SWD is a relatively new design and therefore further research is warranted in order to inform researchers, reviewers and ethical committees better with respect to the question which design to prefer for the study question at hand.

## Abbreviations

CRT: Cluster randomized trial
SWD: Stepped wedge design
ICC: Intracluster correlation coefficient
